# Twisted Bilayer MoS_2_ under Electric Fields: A System with Tunable Symmetry

**DOI:** 10.1021/acs.nanolett.4c04556

**Published:** 2024-12-11

**Authors:** Aitor Garcia-Ruiz, Ming-Hao Liu

**Affiliations:** † Department of Physics and Center for Quantum Frontiers of Research and Technology (QFort), National Cheng Kung University, Tainan 70101, Taiwan; ‡ Department of Physics and Astronomy, University of Manchester, Oxford Road, Manchester, M13 9PL, United Kingdom; ¶ National Graphene Institute, University of Manchester, Booth St. E., Manchester, M13 9PL, United Kingdom

**Keywords:** Twistronics, Artificial lattices, MoS_2_, Quantum transport, Hofstadter’s butterfly, Chern numbers

## Abstract

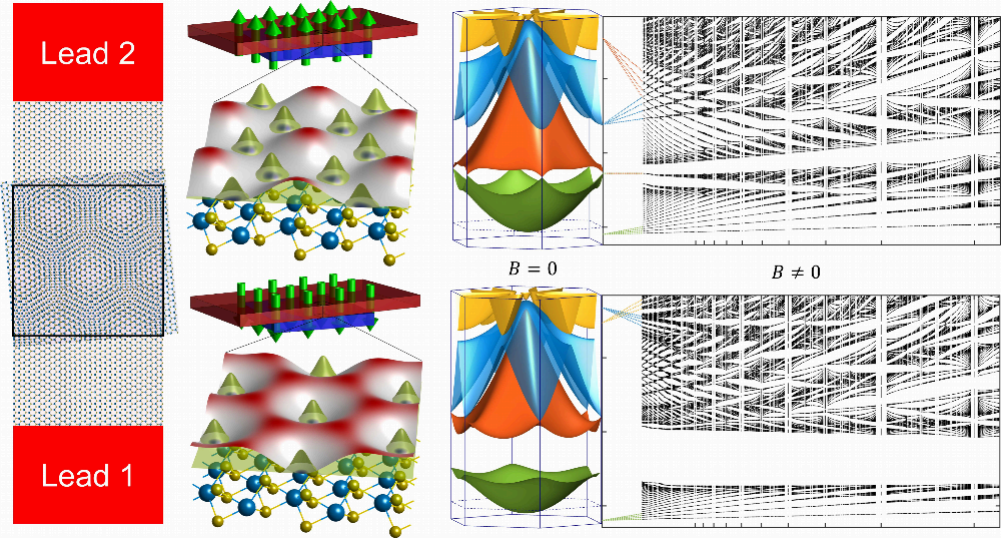

Gate voltages take full advantage of 2D systems, making it possible to explore novel states of matter by controlling their electron concentration or applying perpendicular electric fields. Here, we study the electronic properties of small-angle twisted bilayer MoS_2_ under a strong electric field. We show that transport across one of its constituent layers can be effectively regarded as a two-dimensional electron gas under a nanoscale potential. We find that the band structure of such a system is reconstructed following two fundamentally different symmetries depending on the orientation of the external electric field, namely, hexagonal or honeycomb. By studying this system under magnetic fields, we demonstrate that this duality not only translates into two different transport responses but also results in having two different Hofstadter’s spectra. Our work opens up a new route for the creation of controllable artificial superlattices in van der Waals heterostructures.

Gate voltages are essential device components.^1^ By controlling the electron concentration of semiconductors, they make transistors possible, which are at the cornerstone of today’s technology.^2^ From the point of view of material science, however, the use of gates extends beyond tuning the Fermi level. During the last decades, scientists have applied this concept to two-dimensional (2D) systems like those composed of graphene or transition-metal dichalcogenides (TMDs) to gain access to a rich variety of phases, including superconductivity,^3,4^ charge density waves,^5^ or ferromagnetism,^6−8^ to name just a few. Here, gate voltages can fully exploit their 2D nature, not only by changing the Fermi level but also applying perpendicular electric fields. Among this family of materials, MoS_2_, a 2D semiconductor with a direct band gap of ∼1.8 eV,^9,10^ has stood up as a candidate to lead the next generation of electronic devices, due to its potential uses as a gas sensor,^11,12^ for flexible low-power consumption devices,^13^ and its integrability as transistors and logic gates in electronic circuits.^14−16^ Furthermore, MoS_2_ is very susceptible to gate voltages, which can tune its concentration^17^ or even induce superconductivity.^18^

On the other hand, nanoscale potentials on 2D materials have become a new paradigm in condensed matter for exploring different phases of matter, opening new avenues to manipulate the electronic, optical, excitonic, thermal, and mechanical properties of the system.^19−35^ These are typically generated by intentionally rotating slightly the crystallographic axis of one of the layers, which introduces a nanometer-scale, moiré interlayer potential. Alternatively, one can engineer a superlattice potential embedded in the substrate by patterning the substrate to achieve a controllable artificial superlattice.^36−43^ In both cases, the nanometer-scale potential also transforms the response of the system to weak magnetic fields (∼1 T), which also introduces a nanometer-scale order, the magnetic length. As a result, the typical discrete spectrum of Landau levels (LL) turns into an intricate self-similar fractal-like structure, often referred to as *Hofstadter’s butterfly*,^44^ which has been studied both theoretically^45−53^ and experimentally.^54−59^

In this Letter, we study the transport across the top layer of a small-angle twisted bilayer MoS_2_ device, rotated with respect to the parallel (AA) configuration (see Figure 1). This system is characterized by a moiré interlayer potential of lattice constant **R**_*m*_ = *a*/2 sin(θ/2), where *a* ≈ 3.18 Å^60−63^ is the lattice constant of MoS_2_ and θ is a small relative angle mismatch between the two layers. This material is susceptible to strong out-of-plane electric fields. Some first-principles calculations even suggest that the Stark effect induced by such electric field can close the band gap between the conduction and valence band, turning the system into a metal.^64−67^ In our case, we propose the application of moderately strong electric fields |**E**| ≈ 0.2 V/nm, which keeps a sizable energy gap between the conduction and valence bands, while inducing an energy shift of ∼100 meV between the two layers, big enough to separate their conduction band edges. Using the relation between electric field and voltage gates |**E**| = (*C*_*bg*_*V*_*bg*_ – *C*_*tg*_*V*_*tg*_)/2ε_0_ε_*r*_, where *C*_*bg*/*tg*_ ∼ 100 nF·cm^–2^ are the typical values for gate voltage capacitance between the bottom/top layers and the sample^68^ and ε_*r*_ ∼ 5 is the relative permittivity of the MoS_2_ monolayer,^69^ one gets the estimate *V* ∼ 10 V for the magnitude of the voltage, which is experimentally achievable. The resulting system can be effectively analyzed as a two-dimensional electron gas (2DEG) on a nanoscale moiré potential, whose Hamiltonian takes the form (see Supporting Information)
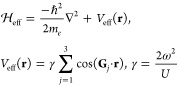
1where *m*_*e*_ ≈ 0.46 *m*_0_(70) is the effective mass of the 2DEG in MoS_2_ (*m*_0_ is the free electron mass), , and ω ∼ 10 meV^65,71−76^ is the coupling between the two monolayers. The on-layer energy shift *U* ≫ ω is introduced by the external electric field, which prevents the direct band anticrossing by spectrally separating the two conduction band edges. The band reconstruction in each layer is then the result of virtual tunneling processes to the other layer, thus making the magnitude and sign of the effective parameter γ controllable by the external electric field.

**Figure 1:**
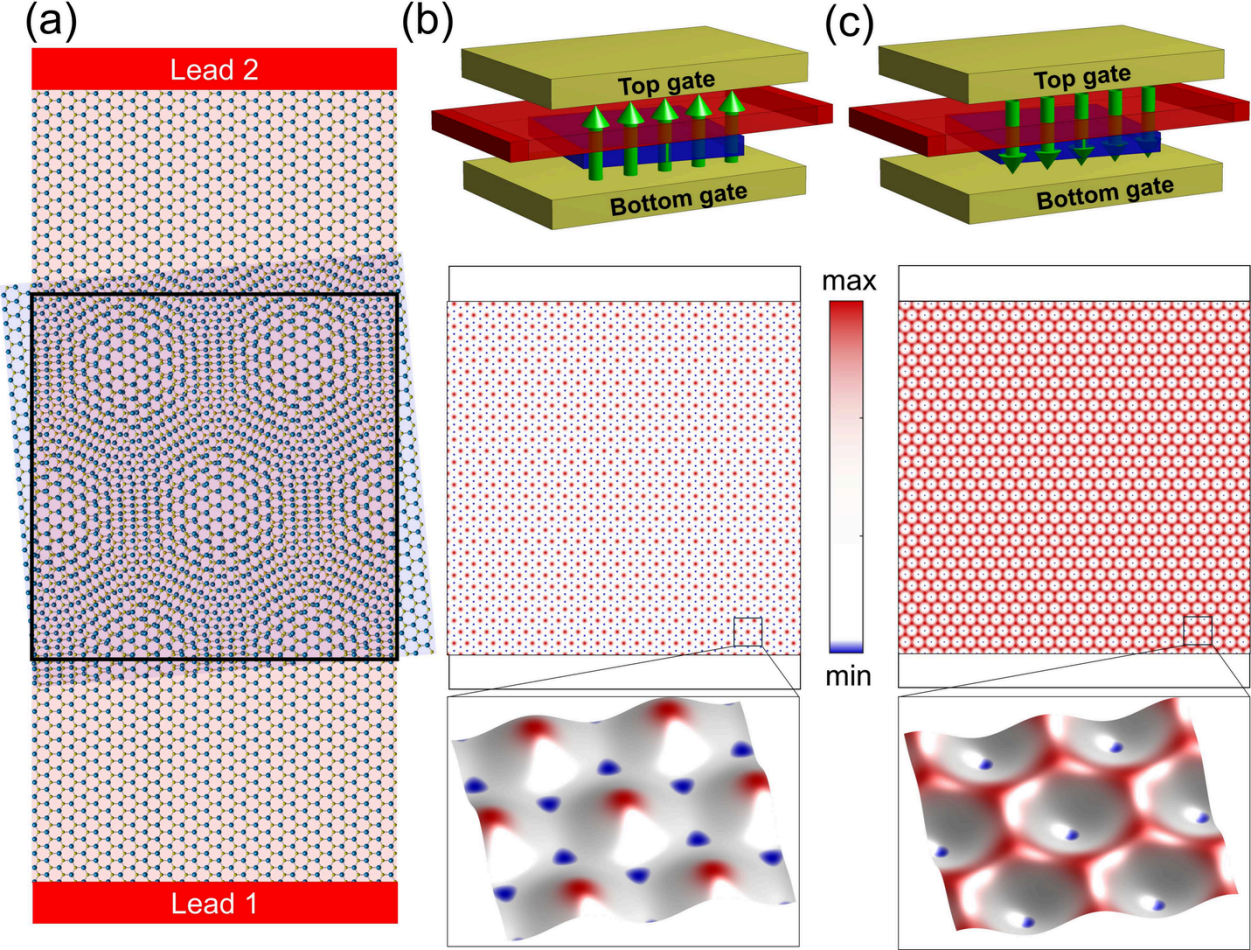
(a) Schematic of a two-terminal MoS_2_ device, with a scattering region where an additional MoS_2_ monolayer is stacked and twisted, which generates a superlattice potential presented in panels on the right. In our quantum transport simulations, we considered a 500 × 500 nm scattering region, with a twist angle of θ = 1° (*R*_*m*_ ≈ 18.5 nm) and a superlattice potential strength γ = 2 meV (α = 0.48). (b) Colormap of the potential *V*(**r**) in the scattering region, when the displacement field has bottom-to-top orientation. The lowest energy valleys of *V*(**r**) form a honeycomb lattice. (c) Same as in (b) but with the opposite orientation of the displacement field. Here, the lowest energy valleys of *V*(**r**) form a hexagonal lattice.

Up to a scaling factor, the miniband spectrum depends on the dimensionless parameter α = γ/*E*_κ_, where  meV is the energy of the conduction band at the corners of the mini Brillouin zone for γ = 0. Here, we can apply the continuum model^27,77−79^ to diagonalize the Hamiltonian in eq 1 and compute numerically the density of states (DoS) using
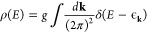
2where *g* is the degeneracy of the bands. In graphene systems, it is usual to take *g* = 4, reflecting the valley and spin degeneracy. In MoS_2_, however, due to the spin–orbit coupling, the conduction band splits into two spin-up and spin-down polarized bands by about 15 meV.^80^ Therefore, we will consider a degeneracy factor of *g* = 2 and used the spin–orbit splitting value to set an upper limit to the twist angle for which our model remains valid: in order to satisfy *E*_κ_ < 15 meV, the twist angle must be θ ≲ 2°. At these low angles, it is known that lattice reconstructions are present in twisted TMDs, but the qualitative features of the first minibands remain unaffected (Supporting Information).

In Figure 2(a), we show two exemplary miniband spectra. For α = −0.48, the lowest miniband becomes spectrally isolated from the rest, resembling the dispersion obtained from a tight-binding model on a hexagonal lattice. This is because the wave function is localized around the minima of *V*(**r**), which for negative values of γ always form an *artificial hexagonal lattice*. In contrast, for α = 0.48, the lowest two minibands exhibit a graphene-like dispersion, with touching points at the corners of the mini-Brillouin zone (mBZ), mimicking the low-energy Dirac-like dispersion of graphene. This resemblance originates from the position at which the low-energy states are localized in real space around the minima of *V*(**r**), forming a honeycomb lattice [see Figure 1 (b)]. This feature is known to emerge in generic 2DEG under hexagonal potentials, which is why it is often referred to as *artificial graphene*.^36,81−83^ In panel (b) of the same figure, we present a gray scale DoS map of the miniband spectra for |α| ≤ 2.3. This map allows us to visualize the role of the sign of α, which prescribes two completely different band reconstructions: for negative values, the first band is always spectrally isolated, while for positive values, we observe a region of low DoS, corresponding to the Dirac point between the first two graphene-like bands, which also become isolated for α ≳ 1.5.

**Figure 2:**
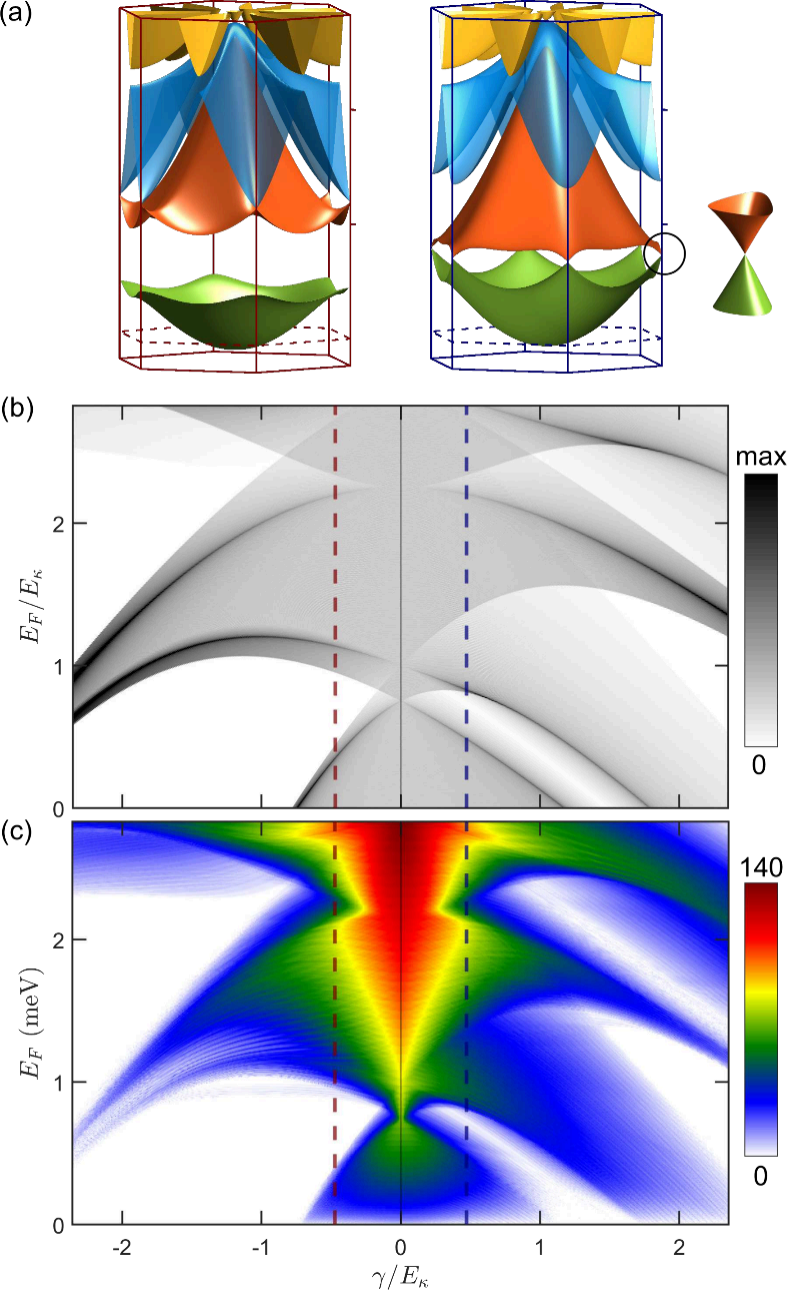
(a) Band structure in the first mini Brillouin zone for α = −0.48 (left) and α = +0.48 (right). (b) Gray-color map of the density of states for values of |α| ≤ 2.3. (c) Quantum transport across the two-terminal device shown in Figure 1(a), also for |α| ≤ 2.3.

The reconstruction of the conduction band edge can be tracked in transport experiments. Here, we simulate the conductance across a realistic two-terminal device, where only the top layer is contacted [see Figure 1(a)]. Following the real-space Green’s function method,^84,85^ we model the parabolic dispersion of MoS_2_ using a tight-binding model on a square lattice, where there is a 500 × 500 nm scattering region characterized by a potential with periodicity 18.5 nm (θ = 1°), and compute the conductance for a range of superlattice coupling strength values of −10 ≤ γ ≤ 10 meV. The Hamiltonian of the system is
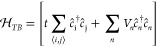
3

Above,  is the operator that annihilates (creates) an electron at the lattice site *i*, ⟨··· ⟩ denotes nearest neighbors, *t* = −*ℏ*^2^/2*m*_*e*_*a*^2^ is the hopping parameter, with *a* = 2 nm being the lattice spacing of our square lattice, and *V*_*n*_ = *V*_*eff*_(***r***_*n*_) inside the scattering region and region on the leads. Note that while our square lattice has a much larger unit cell and does not even have the same symmetry of the MoS_2_ crystal, our parameter choice grants the same low-energy dispersion, which fully determines the properties of ballistic transport. In the same spirit of the scalable tight-binding model^86^ this approach allows us to simulate ballistic conductance in realistic device models without requiring exceedingly demanding computational power. The scattering region is connected to two semi-infinite leads, across which the conductivity is computed using , with *T* being the transmission probability, that is, the sum over all propagating modes across the two leads below the Fermi level,^87^ hence assuming zero-temperature. In panel (c) of Figure 2, we present the transmission across our two-terminal device. The conductance map is consistent with the DoS map computed from the miniband spectrum, where low-density states correlate with low transmission and the insulating signal corresponds to gaps in the dispersion. This observation confirms that transmission also reflects the symmetry duality of the system.

Adding a perpendicular magnetic field, **B** = (0, 0, *B*), induces strong changes in the electronic spectrum. After the Luttinger substitution,^88^ the first term in eq 1 generates a discrete series of LLs with energies *E*_*n*_ = (*n* + 1/2)*ℏω*, where , and ω = *eB*/*m*_0_. The second term couples LLs with guiding centers shifted by an amount Δ, determined by the commensurability condition  (*p*, *q* are co-prime integers and  is the magnetic flux across the nonmagnetic unit cell).^46^ For an explicit derivation of the matrix elements of the Hamiltonian, see Supporting Information. The eigenvalues of the resulting Hamiltonian are used to compute the band structure and DoS, while the eigenvectors encode the information to obtain the Chern numbers associated with each band, which we compute following refs (46) and (89). In the transport calculations, we incorporate the magnetic field in the scattering region by the Peierls substitution,^90^ where the hopping parameter acquires a space-dependent complex phase factor . Note that in order to compute a magnetic band structure, only a discrete set of magnetic fields constrained by the commensurability condition are allowed, whereas in transport simulations, one can tune continuously the value for the magnetic field strength.

The magnetic spectrum for α = −0.48 is shown in Figure 3(a), for values of the magnetic flux up to about one-third of Φ_0_ (*B* = 5θ^2^ T), alongside an exemplary magnetic band structure for *p* = 2, *q* = 1. The first subset of minibands, spectrally isolated from the rest by a trivial gap, originates from the lowest energy band in Figure 1(a), and it corresponds to a portion of the Hofstadter’s butterfly for a hexagonal lattice. At energies *E*_*F*_ ≈ 2*E*_κ_, we find other distinct features, such as a rhomboid mesh-like structure, the result of LL intercrossings. These evolve into larva-like energy windows at higher magnetic fields, which coexist with other butterfly-like structures. Our transport simulations, carried out on a scattering region with θ = 1° and γ = −2 meV to reproduce the same value of α, are fully consistent with these observations. It is worth mentioning that transport inside the gaps is not always zero but a constant value. We investigate its relationship with the topology of magnetic bands by computing the Chern number of each band and verifying that the number of transport modes equals the sum of Chern numbers of all bands below a gap, as shown in the two right panels of Figure 3. A useful way to visualize the nature of the transport inside the scattering region is by plotting the current maps, as shown in panels (c), which were obtained by evaluating the absolute value of the current density operator, |**J**| ∝ |ψ*∇ψ – ψ∇ψ*|, where ψ represents the eigenfunction for the corresponding value of *B* and *E*. These maps showcase the transport inside the LLs, or bulk transport (first two panels), where the entire scattering region is engaged in the conduction, and transport inside topological band gaps, or edge transport, where the different topology between the material and the vacuum enforces conduction along one side of the scattering region (third panel). We also show the current density inside the trivial map, where there are no transport modes, and the sum of Chern numbers of all bands below is zero (last panel). As expected, the wave function amplitude exponentially decays as it tunnels into the scattering region.

**Figure 3:**
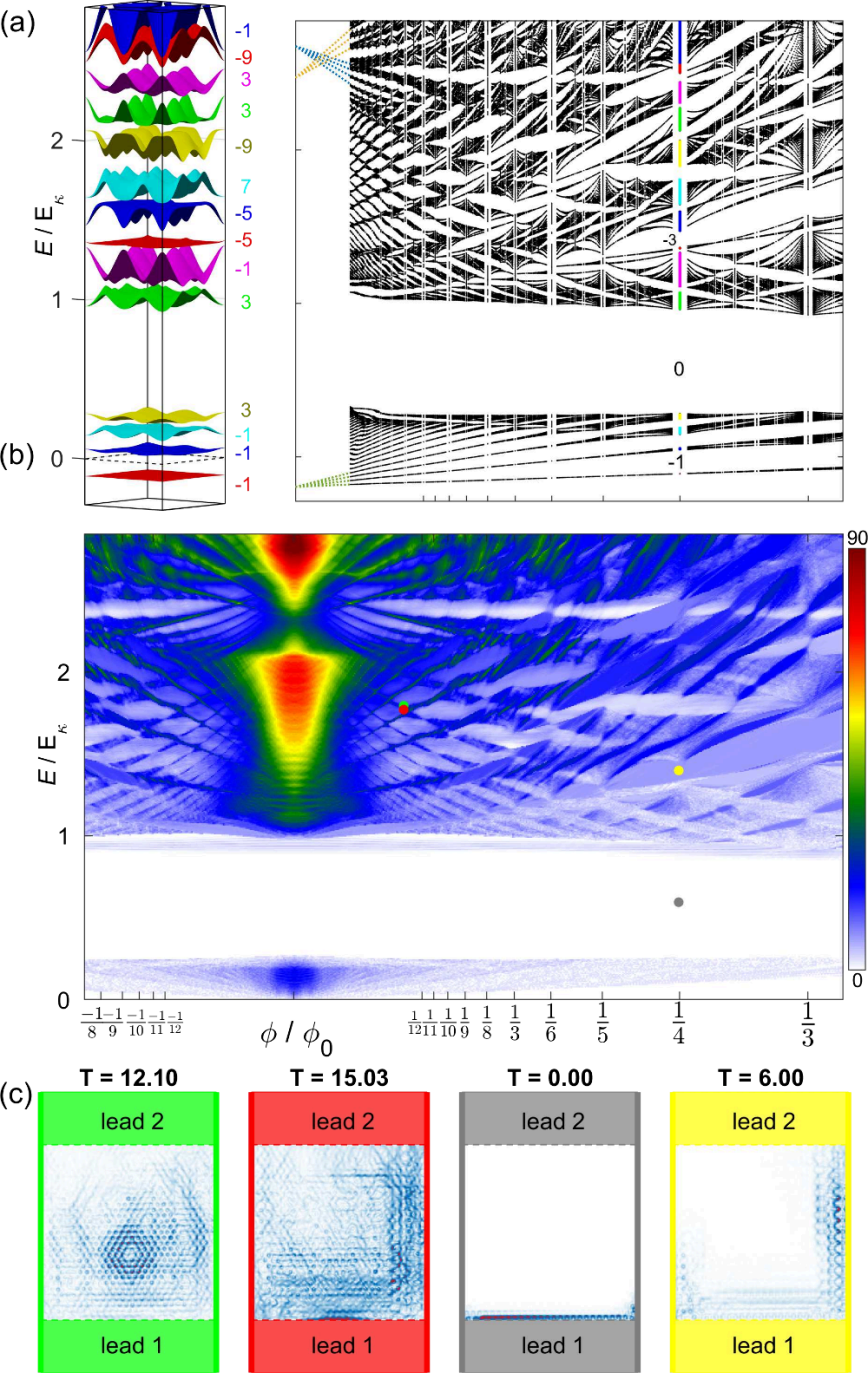
(a) Hofstadter’s butterfly of MoS_2_ on the superlattice potential *V*_eff_(**r**), for α = −0.48 (obtained using *R*_*m*_ = 18.5 nm and γ = −2 meV). On the left, the band structure and the associated Chern numbers for the commensurate structure *p* = 2, *q* = 1 (Φ = Φ_0_/4). (b) Transmission across a two-terminal device on the same system. (c) Current density plots for the four marks in the panel above, showing bulk transport, edge transport, and absence of transport inside topological gaps.

In Figure 4(a), we present the magnetic spectrum for α = 0.48, as well as the magnetic dispersion for *p* = 2, *q* = 1. At low magnetic flux, we expect the spectrum to turn into a series of LLs, linear in magnetic field, and with origin at the parabolic band edges of the nonmagnetic bands in Figure 2(a). While the spectrum shares some commonalities with its α < 0 counterpart, such as a rhomboid-like structure that evolves into larva-shaped energy windows, the magnetic spectrum at low energies is totally different with two band gap windows at *E* ≈ 0.7*E*_κ_ branching from Φ ≈ Φ_0_/25 and separated by the fractal evolution of the zeroth-order LL of the conical dispersion shown in the inset of Figure 2(a). These features are also well-captured by the simulated quantum transport in panel (b) of the same figure, where we also checked that the numerically computed value of the conductance coincides with the sum of the Chern numbers of all the bands below and further confirmed by panel (c), where we show again the bulk and edge transport current density maps.

**Figure 4:**
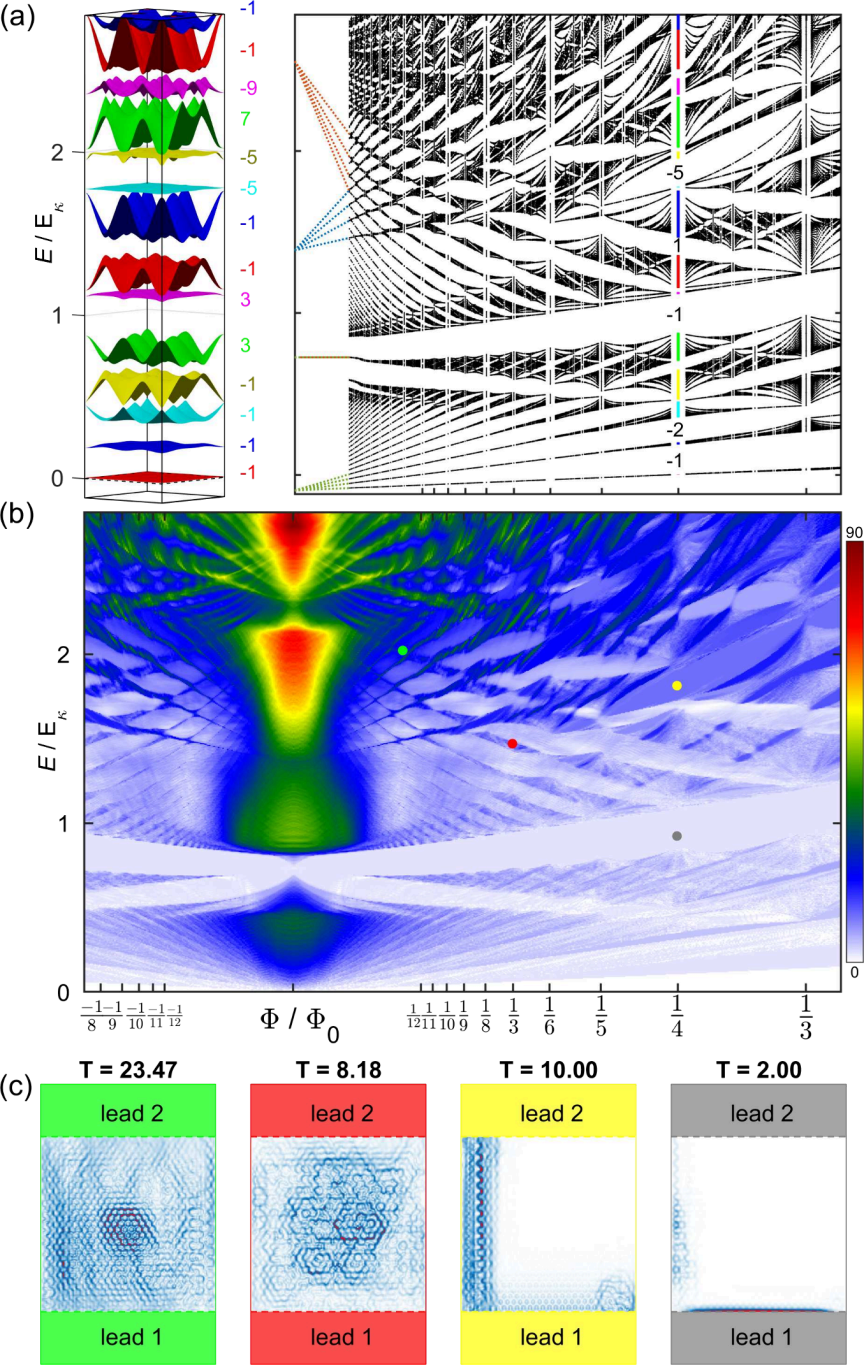
(a) Hofstadter’s butterfly of MoS_2_ on the superlattice potential *V*_eff_(**r**), for α = +0.48 (obtained using *R*_*m*_ = 18.5 nm and γ = 2 meV). On the left, the band structure and the associated Chern numbers for the commensurate structure *p* = 2, *q* = 1 (Φ = Φ_0_/4). (b) Transmission across a two-terminal device on the same system. (c) Current density plots for the four marks in the panel above, as in Figure 3, showing bulk transport and edge transport. This time, all gaps are nontrivial, and the conductance inside them is exactly the number of edge states multiplied by the fundamental conductance 2*e*^2^/*h*.

The presence of nontrivial gaps for values of the dimensionless parameter for 0 < α < 1.5 should produce nonzero conductance across the entire transmission map, in contrast to its α < 0 counterpart. Because this should provide a clear experimental signature in transport experiments, in Figure 5 we also present the Wannier diagram for θ = 1° and γ = 2 meV. This plot is constructed by broadening the Hofstadter’s butterfly of Figure 3 and Figure 4 with a Lorentzian of width *E* ∼ 0.1 meV and presenting it as a function of doping and magnetic field. As expected, both diagrams have many features in common, with a series of LLs for low magnetic fields and low doping levels coming from the parabolic band edges, Brown–Zak oscillations *B* ≈ 14/*n* T (see Supporting Information for details). The larva-like structures in the Hofstadter’s spectrum generate discontinuous lines of empty states in the top-left portion of the diagram in both cases. However, the diagram for γ = −2 meV also shows a horizontal line of empty states at *n* ≈ 6.7 × 10^11^cm^–2^, where the lowest-energy set of minibands are filled and completely separated from the rest by a trivial gap. This observation is robust, as the size of the trivial gap is about 2 meV and only appears for one orientation of the external electric field applied, and therefore it can be used for sample calibration.

**Figure 5:**
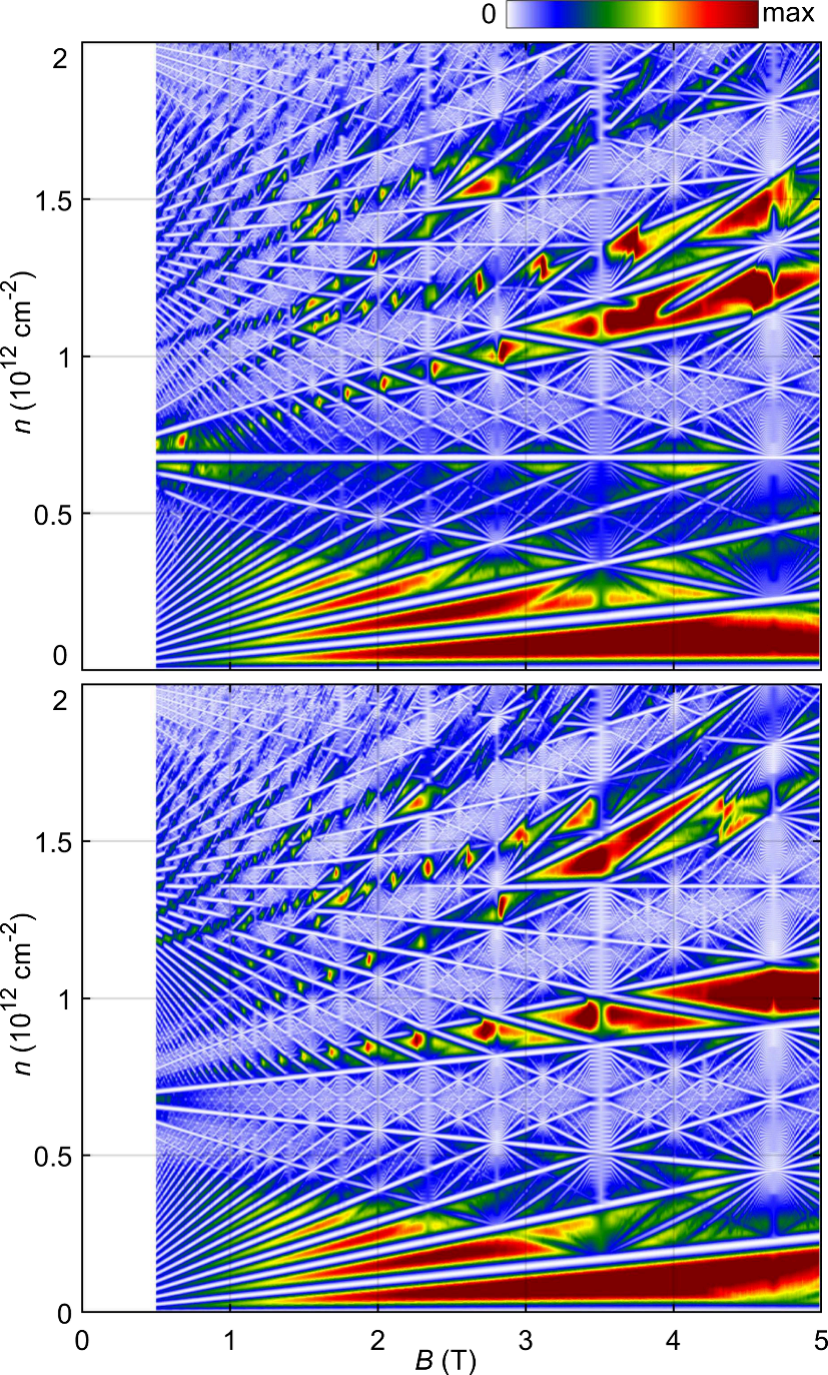
Wannier diagram for γ = −2 meV (top) and γ = 2 meV (bottom). In both cases, the intermittent white lines at *B* ∼ 1 T and *n* ≈ 1.5 × 10^12^ cm^–2^ are a signature of the mesh-like patterns in the Hofstadter’s butterfly. The horizontal line of empty states at *n* ≈ 6.7 × 10^11^ in the top panel marks the filling of the first set of magnetic minibands, for which the material is a trivial insulator.

Overall, our research proposes a way of creating an artificial lattice with controllable symmetry on 2DEG using MoS_2_, capable of turning a trivial insulator into a semimetal with graphene-like properties by inverting the orientation of an external electric field. This dichotomy extends to their distinct magnetic responses, developing two different Hofstadter’s butterflies, and can be traced in transport experiments. Our results are applicable to twisted bilayer MoS_2_, with angles between θ ∼ 1° and 2°. Below these values, the relaxation effects^91^ as well as the domain asymmetric expansion induced by the electric field^92^ cannot be ignored. Here, we have ignored defects, which could broaden the peaks in the transport maps,^93−95^ and phonons. We also estimated the temperature at which acoustic phonons in MoS_2_ start enabling Umklapp scattering^96,97^ (*T* ≈ *s*·*G*_*m*_/*k*_*B*_ ∼ 0.1 K, with *s* ≈ 2 meV·nm^98^ being the velocity of sound for phonons and *k*_*B*_ the Boltzmann constant) and concluded that the addition of phonons in the model would simply add some imaginary component of the self-energy of electrons, leading to a broadening of their spectrum.

## Supporting Information Available

(i) Derivation for the effective Hamiltonian employed in eq 1 from the two-MoS_2_ layer model, (ii) band-integrated amplitude of the wave functions for the low-energy bands, (iii) theoretical description of the method used to compute the Hofstadter’s butterfly in the framework of the continuum model, and (iv) effects of lattice relaxation on the band structure (PDF)
