# Supplementary material for: Twisted Bilayer MoS2 under Electric Fields: A System with Tunable Symmetry
Source: Nano Lett. 2024 Dec 11;24(51):16317–24. doi: 10.1021/acs.nanolett.4c04556 (PMC11673579; doi:10.1021/acs.nanolett.4c04556)
Supplement: Supplementary file 1 — nl4c04556_si_001.pdf [file nl4c04556_si_001.pdf]

Twisted Bilayer MoS_2 Under Electric Fields: a System with Tunable Symmetry

Aitor Garcia-Ruiz*,† and Ming-Hao Liu*,†

†*Department of Physics and Center for Quantum Frontiers of Research and Technology (QFort), National Cheng Kung University, Tainan 70101, Taiwan*

‡*Department of Physics and Astronomy, University of Manchester, Oxford Road, Manchester, M13 9PL, United Kingdom*

¶*National Graphene Institute, University of Manchester, Booth St. E., Manchester, M13 9PL, United Kingdom*

E-mail: aitor.garcia-ruiz@phys.ncku.edu.tw; minghao.liu@phys.ncku.edu.tw

Effective Hamiltonian

Twisted bilayer MoS_2 has been extensively analysed as a joint structure of two equally relevant monolayers of TMD, due to their potential to engineer topological insulators, flat bands or skyrmion lattice textures[1–5]. Here, we also consider the effect of a strong electric field, which overshadows any other source of intralayer potential by shifting the on-site energy of the bottom layer. Then, by plotting the resulting band structure projected to the top layer, we demonstrate the validity of Eq. (1) in the main text.

Our focus is on the reconstruction of the conduction band edges of the MoS_2, which are located at the corners of the Brillouin zone and mostly composed of the d-orbitals of the Mo atoms[6–9]. When two MoS_2 monolayers are stacked on top of each other and twisted with respect to the AA-configuration, as shown in Fig. S1 (a), the global minimum of the first

conduction band is still located at the K point[10], and the effective Hamiltonian of the system is given by

$$\begin{pmatrix} H^t(\mathbf{k}) & V(\mathbf{r}) \\ V^\dagger(\mathbf{r}) & H^b(\mathbf{k}) - U \end{pmatrix} \begin{pmatrix} \Psi^t \\ \Psi^b \end{pmatrix} = E \begin{pmatrix} \Psi^t \\ \Psi^b \end{pmatrix}, \tag{1}$$

$$H^{t/b}(\mathbf{k}) = \frac{\hbar^2}{2m_e}|\mathbf{k} \mp \xi\mathbf{q}_0/2|^2, \qquad V(\mathbf{r}) = \omega \sum_{\mathrm{j}=0}^{2} e^{i\xi\mathbf{q}_\mathrm{j}\cdot\mathbf{r}}.$$

Above, $\xi = \pm 1$ stands for the valley index, the vectors $\mathbf{q}_\mathrm{j} = q\left[-\sin(\frac{2\pi}{3}\mathrm{j}), \cos(\frac{2\pi}{3}\mathrm{j})\right]$, with $q = 8\pi\sin(\theta/2)/3a$, are the wavenumber mismatch between the corners of the Brillouin zone of MoS_2, $\omega \sim 10$ meV[1] is an effective coupling parameter between the two monolayers and U is the energy shift produced by the electric field. To show that this Hamiltonian is approximately equivalent to that of Eq. (1) of the main text, we diagonalize it using the continuum model, taking $\omega = 10$ meV, $\theta = 1^\circ$ and values of the on-site energy U spanning from -100 to 100 meV. The resulting band structure present the band structure in Fig. S1 (b), where we centre the mini Brillouin zone at the κ point and each point in the band structure is assigned a value of the opacity proportional to its top-layer projection, to highlight the similarities with those in Fig. 2 of the main text.

To understand the observation above, we project the Hamiltonian in Eq. (1) on the top layer. First, we express Ψ^b in terms of Ψ^t from the second line in Eq. (1), and replace it in the first line,

$$H^t(\mathbf{k})\Psi^t + V(r)[E - H^b(\mathbf{k}) + U]^{-1}V^\dagger(r)\Psi^t = E\Psi^t. \tag{2}$$

We are interested on energies close to the conduction band edge of the top layer, which we set at $E = 0$. On the other hand, for strong electric fields, $U \gg H^{t/b}(\mathbf{k})$ is expected to be the largest energy scale, which entitles us to approximate the operator $[E - H^b(\mathbf{k}) + U]^{-1} \approx 1/U$.

This provides the effective Hamiltonian for the top layer

$$\mathcal{H}_{\rm eff} = H^t(\mathbf{k}) + \frac{\omega^2}{U}\sum_{\rm j',j} e^{i\mathbf{q}_{\rm j'}\cdot\mathbf{r}} e^{i\mathbf{q}_{\rm j}\cdot\mathbf{r}}$$
$$= H^t(\mathbf{k}) + \frac{3\omega^2}{U} + \frac{2\omega^2}{U}\left\{\cos[(\mathbf{q}_1-\mathbf{q}_0)\mathbf{r}] + \cos[(\mathbf{q}_2-\mathbf{q}_1)\mathbf{r}] + \cos[(\mathbf{q}_0-\mathbf{q}_3)\mathbf{r}]\right\}, \qquad (3)$$

which is formally the same as Eq. (1) of the main text for $\gamma = 2\omega^2/U$. To note, the sign of the effective parameter $\gamma = 2\omega/U$ can be controlled by the orientation of the external electric field, which results in two utterly contrasting band reconstructions.

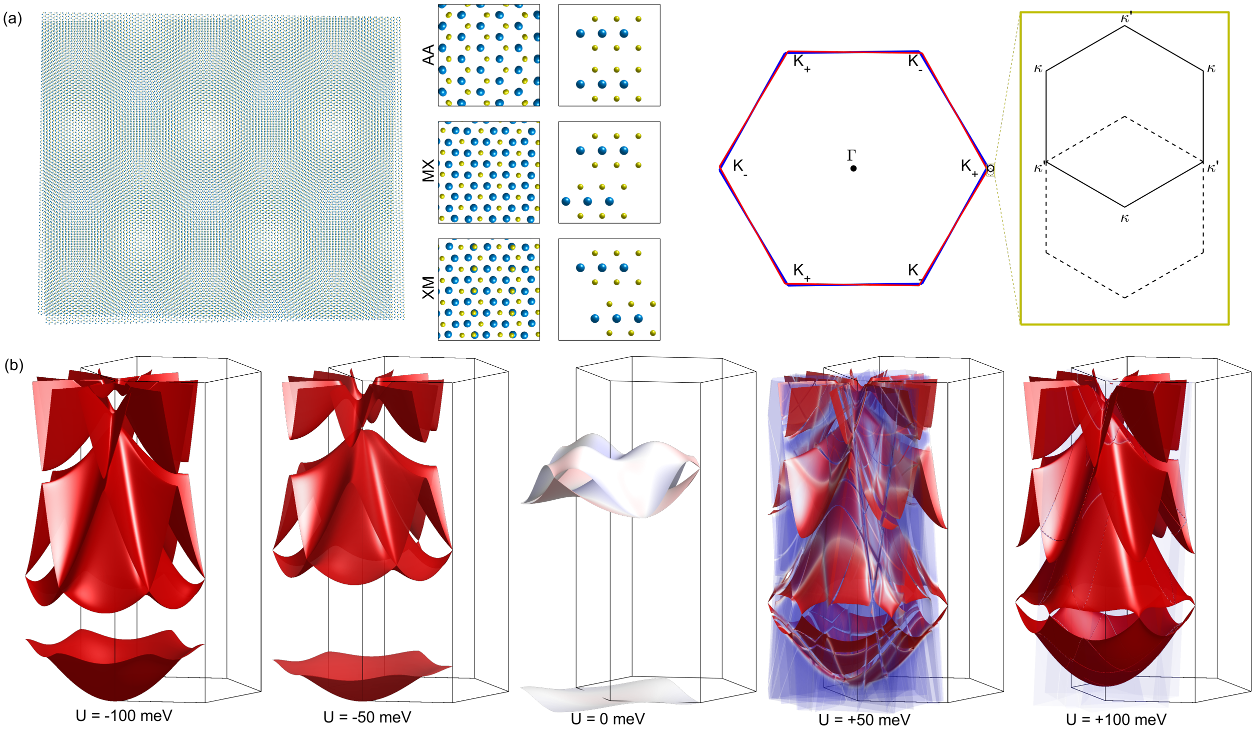

Figure S1: (a) Pictorial representation of twisted bilayer MoS_2, highlighting the three types of stacking across the superlattice, AA, MX and XM. On the right, the Brillouin zone of both MoS_2 monolayers, and the mini Brillouin zone around the $\mathbf{K}_+$ valley in solid black lines. We present the band structure in dashed black lines, for a better comparison with the band structures in Fig. 2 of the main text. (b) From left to right, band structure for $U = -100, -50, 0, +5$ and $+100$ meV, where we took $\omega = 10$ meV. The level of opacity is proportional to the wavefunction composition on the top layer.

Spatial localization of wavefunctions in the first minibands.

In this section, we present the spatial charge distribution of states for the non-magnetic miniband spectrum of the conduction band edge of MoS_2 under the effective superlattice potential in Eq. (3) $V(\mathbf{r})$. Following previous works[11], we compute the squared amplitude of the eigenvector

$$\Phi^b(\mathbf{k}, \mathbf{r}) = \frac{1}{\sqrt{N}} \sum_{\mathbf{G}} c^b_{\mathbf{G}}(\mathbf{k}) e^{i(\mathbf{k}-\mathbf{G})\cdot\mathbf{r}}, \tag{4}$$

where the the term $c^b_{\mathbf{G}}(\mathbf{k})$ refers to the component of the wavefunction associated to the Bloch function shifted by $\mathbf{G} = a_1\mathbf{G}_1 + a_2\mathbf{G}_2$, in the b-th band at the point $\mathbf{k}$. We demonstrate numerically that the lowest energy states localize around the minima the superlattice potential by integrating the absolute squared amplitude of the expression above over the first mini Brillouin zone for each miniband,

$$n^b(\mathbf{r}) = g \int_{\text{mBZ}} \frac{d\mathbf{k}}{(2\pi)^2} \left| \sum_{\mathbf{G}} c^b_{\mathbf{G}}(\mathbf{k}) e^{-i\mathbf{G}\cdot\mathbf{r}} \right|^2 \tag{5}$$

In Fig. S2, we plot next to each miniband the integrated charge distribution $n^b(\mathbf{r})$ for $\gamma = \pm 6$ meV. For $\gamma = 6$ meV, the first two bands localize around the minima of $V(\mathbf{r})$, thus forming a honeycomb lattice, while the third band features hot spots of electronic amplitude in the mid-points of these minima, which form a Kagome lattice. Conversely, for $\gamma = -6$ meV, the minima of the superlattice potential where the lowest energy states localize form a hexagonal lattice.

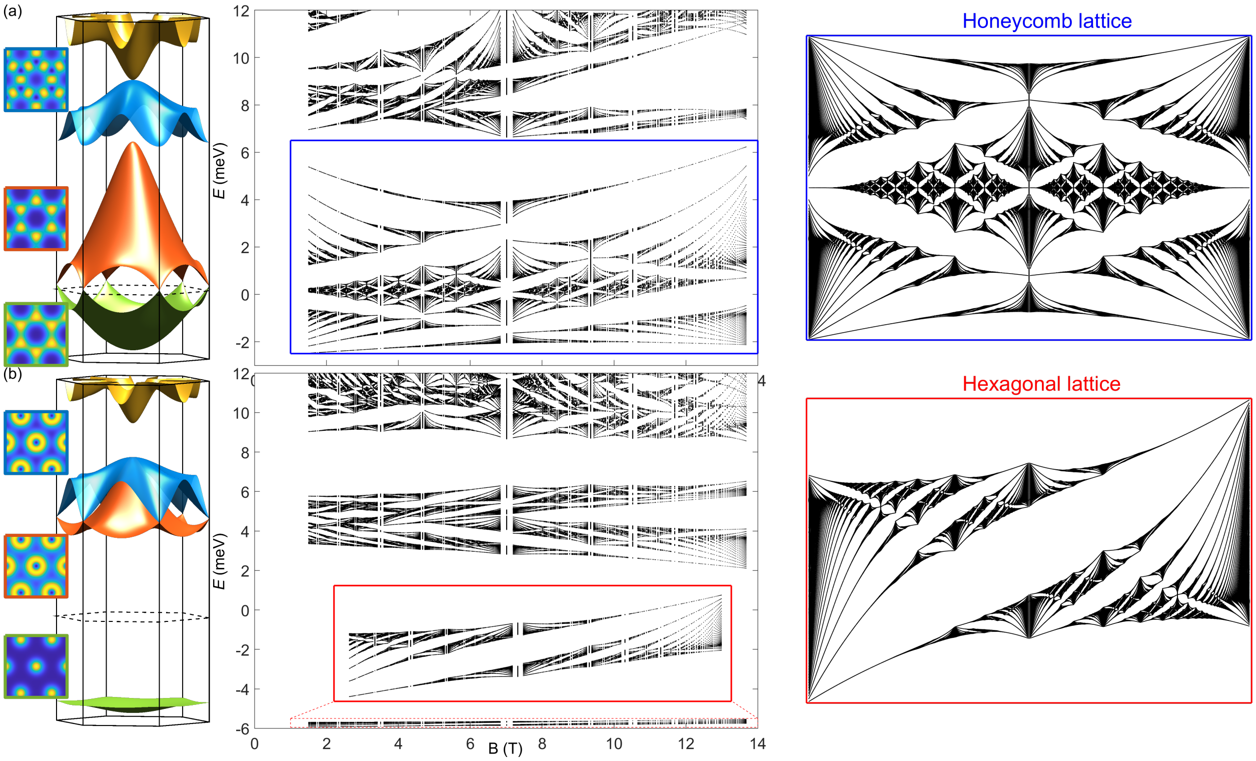

Figure S2: (a) Miniband structure of the conduction band edge of MoS_2 for $\gamma = 6$ meV. Next to each band, we present the integrated spatial charge distribution. The states of the lowest two bands localize around the minima of the superlattice potential, which form a honeycomb lattice. On the right, we show the corresponding Hofstadter's spectrum. The lowest part of the diagram, highlighted in blue, resembles the Hofstadter's butterfly of graphene. In (b) we present a similar analysis with $\gamma = -6$ meV

Magnetic band structure and Hofstadter's butterfly spectrum.

In this section, we expand on the method used to compute the Hofstadter's butterfly and the mini-band spectrum shown in Figs. (3) and (4) of the main text. The spectrum of the valence and conduction band edges of MoS_2 under the effects of a magnetic field, turns into a series of perfectly flat bands, known as Landau levels[12]. Using the Landau gauge $\mathbf{A} = (-yB, 0, 0)$, the eigenvalues and eigenvectors take the form

$$E_{\beta,n} =(n+1/2)\hbar\omega_\beta, \qquad \omega_\beta = \frac{eB}{m_\beta}, \tag{6a}$$

$$|n, y_0\rangle =\frac{1}{\sqrt{L_x}} e^{ik_x x} \cdot \psi_n\left(\frac{y-y_0}{l_B}\right), \qquad l_B = \sqrt{\frac{\hbar}{m|\omega_\beta|}}, \qquad y_0 = k_x l_B^2 \tag{6b}$$

where $\psi_n(x) = \left(2^n n! \sqrt{\pi}\right)^{-1/2} H_n(x)\, e^{-\frac{x^2}{2}}$, and $H_n(x)$ are the Hermite polynomials[13]. As shown below, the periodic potential can couple two eigenvectors with guiding centres differing by a to-be-determined amount Δ. We use the basis of unperturbed Landau levels in Eqs. (6) to construct a larger (magnetic) unit cell by grouping together q number of eigenvectors,

$$\{|n, y_0\rangle, |n, y_0+\Delta\rangle, |n, y_0+2\Delta\rangle, \cdots, |n, y_0+(q-1)\Delta\rangle\}, \tag{7}$$

with $|n, y_0+\Delta q\rangle$ being equivalent to $|n, y_0\rangle$, and the number $q \in \mathbb{N}$ is the number of Landau levels in the magnetic unit cell. Assuming our sample contains M unit cells, we introduce the magnetic Bloch functions,

$$|n, y_0, j, k_y\rangle = \frac{1}{\sqrt{M}} \sum_{m=0}^{M} e^{i(mq+j)\Delta k_y} |n, y_0 + (mq+j)\Delta\rangle, \tag{8}$$

and use them to evaluate the matrix elements of the Hamiltonian,

$$\langle n', y_0', j', k_y'|\mathcal{H}|n, y_0, j, k_y\rangle = E_n^{\beta}\delta_{n',n}\delta_{y_0',y_0}\delta_{j',j}\delta_{k_y',k_y} + \frac{\gamma}{2}\sum_{j=1}^{3}\langle n', y_0', j', k_y'|e^{i\mathbf{G}_j\cdot\mathbf{r}} + e^{-i\mathbf{G}_j\cdot\mathbf{r}}|n, y_0, j, k_y\rangle. \tag{9}$$

The integration over the spatial coordinate x in the second term of the expression above fixes the value for Δ, while integration over the spatial coordinate y gives the values for the magnetic field that ensures a commensurate structure. To illustrate this, we compute explicitly the contribution to the matrix elements from the first exponential term,

$$\begin{aligned}
&\langle n', y_0', j', k_y'|e^{i\mathbf{G}_1\cdot\mathbf{r}}|n, y_0, j, k_y\rangle = \langle n', y_0', j', k_y'|e^{i\frac{\sqrt{3}}{2}Gx}e^{i\frac{1}{2}Gy}|n, y_0, j, k_y\rangle \\
&= \frac{1}{M}\sum_{m',m} e^{-i(m'q+j')\Delta k_y}e^{i(mq+j)\Delta k_y}\int_{-\infty}^{\infty} dx\frac{e^{i\left(\frac{\sqrt{3}}{2}G+k_x-k_x'\right)x}}{L_x} \\
&\times \int_{-\infty}^{\infty} dy e^{i\frac{1}{2}Gy}\psi_{n'}\left(\frac{y-y_0-(m'q+j')\Delta}{l_B}\right)\psi_n\left(\frac{y-y_0-(mq+j)\Delta}{l_B}\right) \\
&= \frac{1}{M}\sum_{m',m} e^{-i[(m'-m)q+j'-j]\Delta k_y}\delta_{k_x',k_x+\frac{\sqrt{3}G}{2}}e^{i\frac{1}{2}Gy_0}e^{i\frac{1}{2}Gq\Delta m}e^{i\frac{1}{2}Gj\Delta} \\
&\times \int_{-\infty}^{\infty} d\tilde{y}e^{i\frac{1}{2}G\tilde{y}}\psi_{n'}\left(\frac{\tilde{y}-[(m'-m)q+j'-j]\Delta}{l_B}\right)\psi_n\left(\frac{\tilde{y}}{l_B}\right),
\end{aligned} \tag{10}$$

where the Kronecker delta gives the value for the change in guiding centre, $\Delta = \frac{\sqrt{3}}{2}Gl_B^2$, and the complex exponential term, together with summation over m and m', cancels out unless $\frac{1}{2}Gq\Delta$ is a integer multiple of 2π. Using relations in Eq. (6) we obtain the condition for a commensurable structure

$$\frac{p}{q} = \frac{G\Delta}{4\pi} = \frac{h/e}{\sqrt{3}R_m^2 \times B} \equiv \frac{\Phi_0}{2\Phi}. \tag{11}$$

Above, Φ_0 is the fundamental unit of magnetic flux and Φ is the magnetic flux across one unit cell. Changing the variables $\tilde{y} = \bar{y} + \Delta/2$, Eq. (12) reduces to

$$\langle n', y_0', j', k_y' | e^{i\mathbf{G}_1\cdot\mathbf{r}} | n, y_0, j, k_y\rangle = \frac{1}{M}\sum_{m',m} e^{-i\Delta k_y} e^{i\frac{1}{2}Gy_0} e^{i\frac{1}{2}Gj\Delta} e^{i\frac{1}{4}G\Delta} \delta_{y_0',y_0}\delta_{m'q+j',mq+j+1} \times \int_{-\infty}^{\infty} d\bar{y} e^{i\frac{1}{2}G\bar{y}} \psi_{n'}\left(\frac{\bar{y}-\Delta/2}{l_B}\right)\psi_n\left(\frac{\bar{y}+\Delta/2}{l_B}\right) \equiv \hat{\mathcal{T}}_j^{(1)} \tag{12}$$

We proceed similarly with the contributions from the other two reciprocal lattice vectors,

$$\langle n', y_0', j', k_y' | e^{i\mathbf{G}_2\cdot\mathbf{r}} | n, y_0, j, k_y\rangle = \frac{1}{M}\sum_{m',m} e^{i\Delta k_y} e^{i\frac{1}{2}Gy_0} e^{i\frac{1}{2}Gj\Delta} e^{-i\frac{1}{4}G\Delta} \delta_{y_0',y_0}\delta_{m'q+j',mq+j+1} \times \int_{-\infty}^{\infty} d\bar{y} e^{i\frac{1}{2}G\bar{y}} \psi_{n'}\left(\frac{\bar{y}+\Delta/2}{l_B}\right)\psi_n\left(\frac{\bar{y}-\Delta/2}{l_B}\right) \equiv \hat{\mathcal{T}}_j^{(2)} \tag{13}$$

$$\langle n', y_0', j', k_y' | e^{i\mathbf{G}_3\cdot\mathbf{r}} | n, y_0, j, k_y\rangle = e^{-iGy_0} e^{-iG\Delta j} \delta_{y_0',y_0}\delta_{j',j} \int_{-\infty}^{\infty} d\bar{y} e^{-iG\bar{y}} \psi_{n'}\left(\frac{\bar{y}}{l_B}\right)\psi_n\left(\frac{\bar{y}}{l_B}\right) \equiv \hat{\mathcal{T}}_j^{(3)}. \tag{14}$$

The integrals over y in the equations above are usually expressed in terms of generalized Laguerre polynomials to ease the numerical procedure[14,15] as

$$\int dy e^{iG_y y} \psi_{n'}\left(\frac{y}{l_B} - \frac{G_x l_B}{2}\right)\psi_n\left(\frac{y}{l_B} + \frac{G_x l_B}{2}\right) = \begin{cases} \left[\frac{(-G_x+iG_y)l_B}{\sqrt{2}}\right]^{n'-n} \sqrt{\frac{n'!}{n!}}\mathcal{L}_n^{n'-n}\left(\frac{G^2 l_B^2}{2}\right) e^{-\frac{G^2 l_B^2}{4}} & n' \geq n \\ \left[\frac{(G_x+iG_y)l_B}{\sqrt{2}}\right]^{n-n'} \sqrt{\frac{n!}{n'!}}\mathcal{L}_{n'}^{n-n'}\left(\frac{G^2 l_B^2}{2}\right) e^{-\frac{G^2 l_B^2}{4}}, & n' < n \end{cases} \tag{15}$$

The matrix elements above allows us to compute the magnetic band structure numerically, setting the maximum the number of Landau levels N_c that ensures convergence in the energy

range of interest. Using the basis of magnetic Bloch functions

$$\Psi_{\boldsymbol{k}} = \begin{pmatrix} |0, y_0, j = 0, k_y\rangle \\ |1, y_0, j = 0, k_y\rangle \\ \vdots \\ |N_c, y_0, j = 0, k_y\rangle \\ |0, y_0, j = 1, k_y\rangle \\ \vdots \\ |N_c, y_0, j = q-1, k_y\rangle \end{pmatrix}, \tag{16}$$

the matrix form of the Hamiltonian, diagonal in the magnetic momentum $k_x = y_0/l_B^2$ and k_y, reads

$$\mathcal{H} = \begin{pmatrix} \hat{E}_n^{\beta} + \frac{\gamma}{2}\left(\hat{\mathcal{T}}^{(3)}_{j=0} + \hat{\mathcal{T}}^{(3)\dagger}_{j=0}\right) & \frac{\gamma}{2}\left(\hat{\mathcal{T}}^{(1)\dagger}_{j=0} + \hat{\mathcal{T}}^{(2)}_{j=1}\right) & 0 & \cdots & \frac{\gamma}{2}\left(\hat{\mathcal{T}}^{(1)}_{j=q-1} + \hat{\mathcal{T}}^{(2)\dagger}_{j=0}\right) \\ \frac{\gamma}{2}\left(\hat{\mathcal{T}}^{(1)}_{j=0} + \hat{\mathcal{T}}^{(2)\dagger}_{j=1}\right) & \hat{E}_n^{\beta} + \frac{\gamma}{2}\left(\hat{\mathcal{T}}^{(3)}_{j=1} + \hat{\mathcal{T}}^{(3)\dagger}_{j=1}\right) & \frac{\gamma}{2}\left(\hat{\mathcal{T}}^{(1)\dagger}_{j=1} + \hat{\mathcal{T}}^{(2)}_{j=2}\right) & \cdots & 0 \\ 0 & \frac{\gamma}{2}\left(\hat{\mathcal{T}}^{(1)}_{j=1} + \hat{\mathcal{T}}^{(2)\dagger}_{j=2}\right) & \ddots & \ddots & 0 \\ \vdots & \vdots & \ddots & \ddots & \vdots \\ \frac{\gamma}{2}\left(\mathcal{T}^{(1)\dagger}_{j=q-1} + \mathcal{T}^{(2)}_{j=0}\right) & 0 & 0 & \cdots & \hat{E}_n^{\beta} + \frac{\gamma}{2}\left(\mathcal{T}^{(3)}_{j=q-1} + \mathcal{T}^{(3)\dagger}_{j=q-1}\right) \end{pmatrix} \tag{17}$$

where $\hat{E}_n^{\beta} = E_n \otimes \mathbb{I}_j$ ($\mathbb{I}_j$ is the unit matrix of dimension j). The Hofstadter's butterfly is obtained by plotting the bandwidth of all bands as a function of magnetic field.

As stated in the main text, the Hofstadter's spectrum inherits the symmetry of a honeycomb (hexagonal) lattice for $\gamma > 0$ ($\gamma < 0$). Here, to further illustrate this feature, we plot on the middle panels of Fig. S2 the Hoftadter's spectra associated to the Hamiltonian in Eq. (1) for $\gamma = +6$ and $\gamma = -6$ meV, and next to which we present the Hofstadter's

butterfly for a honeycomb and a hexagonal lattice in panels (a) and (b), respectively. The latter were obtained using a tight binding model including a Peierls phase, for values of the magnetic fields that allows for a commensurate unit cell[16–19]. The similarity of these spectra reassures the symmetry duality of our system. It is worth mentioning that, while Fig. S2 illustrates the main point, we are neglecting the Zeeman effect, which separates the energy levels of electrons with different spins by $\Delta E = g_e \mu_B B$, with $g_e \approx 2$ the Landé factor and $\mu_B \approx 9.3 \cdot 10^{-24}$ T/J the Bohr mnagneton. The maximum value for this energy splitting in our plot ($B = 14$) is about $\Delta E \approx 1.8$ meV.

Effects of lattice relaxation in small-angle twisted bilayer MoS_2

There are infinite number of aligned stacking configurations in TMD homobilayers, depending on the relative spatial shift. In small-angle twisted homobilayers, in principle, all of them are locally present at one point of the unit cell, but not all of the them are equally stable. The most energetically favorable stacking arrangements will naturally expand at the expense of the more energetically costly ones, leading to an interlayer potential that differs significantly from the sinusoidal potential proposed in this work. Thus, it is natural to ask ourselves the validity of our model if at $\theta \sim 1°$ the main message remains valid.

To answer the question above, we designed a potential based on the relaxation features associated to twisted bilayer MoS_2, rotated from a parallel configuration, shown in Ref.[20]. It captures the inflation of energetically preferable stacking configurations, which feature MX or XM stacking configuration, where the sulfur atoms lie directly on top of the Molybdenum atoms of the other layer. For comparison to the rigid rotation, in Fig. S3 (a) and (b), we present the potential landscape of a rigid rotation, $V(\mathbf{r})$ and the relaxed rotation $\tilde{V}(\mathbf{r})$,

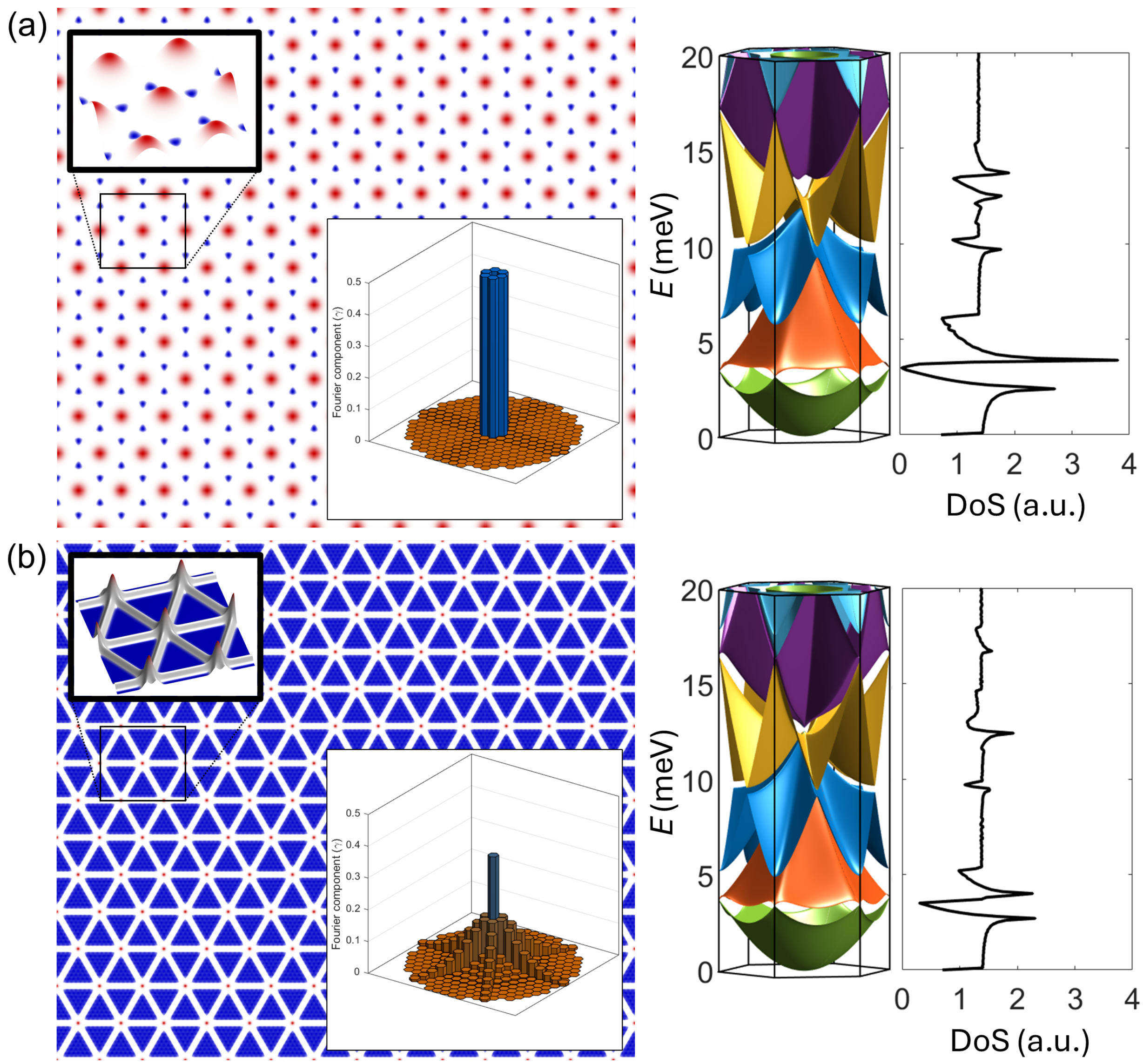

Figure S3: (a) The potential profile in the scattering region of our device, for $R_m = 18.5$ nm and $\gamma = 2$ meV, without considering the effects of relaxation. The bottom right inset highlights the only 6 non-zero Fourier components, in units of γ. On the right, we present the band structure, identical to that of Fig. 2 of the main text, but extended to 20 meV, and the density of states associated. (b) A similar analysis to panel (a) but considering the effects of relaxation. While we observe new peaks in the DoS as a result of the higher order direct coupling, all features become fainter, as the Fourier harmonics are smaller. This indicates that the effect of the superlattice on the dispersion becomes smaller for smaller angles, due to lattice relaxation.

respectively. We obtain numerically the Fourier expansion of the latter,

$$\tilde{V}(\mathbf{r}) = \sum_{\mathbf{G}} V_{\mathbf{G}} e^{i\mathbf{G}\cdot\mathbf{r}}, \tag{18}$$

and plot the Fourier components in the inset panels (b). We incorporate these components in the continuum model as higher order direct couplings, and the resulting band structure, alongside with the Density of states, is presented on the right hand side of panels (a) and (b) of Fig.S3, for rigid and relaxed structures, respectively, for $R_m = 18.5$ nm and $\gamma = 2$ meV. From a qualitative standpoint, both figures present identical general low-energy features, including the formation of graphene-like pair of bands. We observe the band anti-crossing around 18 meV, absent in the rigid counterpart. We expect that at very low angles, the electronic properties are going to be dominated by the domains, and the effect of the superlattice should gradually disappear.

References

[S1] T. Devakul, V. Crepel, Y. Zhang, and L. Fu, "Magic in twisted transition metal dichalcogenide bilayers", Nature Communications, **12**, 6730 (2021).

[S2] F. Wu, T. Lovorn, E. Tutuc, I. Martin, and A. GH. MacDonald, "Topological Insulators in Twisted Transition Metal Dichalcogenide Homobilayers", Physical Review Letters, **122**, 086402 (2019).

[S3] S. Kundu, M. H. Naik, H. R. Krishnamurthy, M. Jain, "Moire induced topology and flat bands in twisted bilayer WSe_2: A first-principles study", Physical Review B, **105**, L081108 (2022).

[S4] Y. Zhang, T. Liu, and L. Fu., "Electronic structures, charge transfer, and charge order in twisted transition metal dichalcogenide bilayers", Physical Review B, **103**, 155142, (2021).

[S5] H. Tang, S. Carr, and E. Kaxiras. "Geometric origins of topological insulation in twisted layered semiconductors", Physical Review B, **104**, 155415, (2021).

[S6] G.-B. Liu, W.-Y. Shan, Y. Yao, W. Yao, and D. Xiao, "Three-band tight-binding model for monolayers of group-VIB transition metal dichalcogenides", Physical Review B, **88**, 085433, (2013).

[S7] S. Fang, R. K. Defo, S. N. Shirodkar, S. Lieu, G. A. Tritsaris, and E. Kaxiras, "Ab initio tight-binding Hamiltonian for transition metal dichalcocagenides", Physical Review B, **92**, 205108, (2015).

[S8] M. Shahriari, A. G. Dezfuli, and M. Sabaeian, "Band structure and orbital character of monolayer MoS2 with eleven-band tight-binding model", Superlattices and Microstructures, **114**, 169 (2018).

[S9] J. Kang, S. Tongay, J. Zhou, J. Li, and J. Wu, "Band offsets and heterostructures of two-dimensional semiconductors", Applied Physics Letters, **102**, 012111, (2013).

[S10] S. Venkateswarlu and A. Honecker, and G. Trambly de Laissardiere, "Electronic localization in twisted bilayer MoS2 with small rotation angle", Physical Review B, **102**, 081103, (2020).

[S11] A. Garcia-Ruiz, J. J. P. Thompson, M. Mucha-Kruczynski, and V. I. Fal'ko, "Electronic Raman Scattering in Twistronic Few-Layer Graphene", Physical Review Letters, 125, 197401 (2020).

[S12] L. Landau, "Diamagnetismus der Metalle", Zeitschrift fur Physik, 64, 629-637 (1930).

[S13] F. W. J. Olver, D. W. Lozier, Ronald F. Boisvert, and C. W. Clark, "NIST handbook of mathematical functions", U.S. Department of Commerce, National Institute of Standards and Technology, Washington, DC; Cambridge University Press, Cambridge (2010).

[S14] R. Bistritzer and A. H. MacDonald, "Moire Bands in Twisted Double-Layer Graphene". Proceedings of the National Academy of Sciences, 108, 12233-12237 (2011).

[S15] K. Hejazi, C. Liu, and L. Balents, "Landau levels in twisted bilayer graphene and semiclassical orbits" Physical Review B, 100, 035115 (2019).

[S16] R. Peierls, "Zur Theorie des Diamagnetismus von Leitungselektronen", Zeitschrift fur Physik, 80, 763-791 (1933).

[S17] J.-W. Rhim and K. Park, "Self-similar occurrence of massless Dirac particles in graphene under a magnetic field", Physical Review B, 86, 235411, (2012).

[S18] J. Li, Y.-F. Wang, and C.-D. Gong, "Tight-binding electrons on triangular and kagome lattices under staggered modulated magnetic fields: quantum Hall effects and Hofstadter butterflies", Journal of Physics: Condensed Matter, 23, 156002 (2011).

[S19] G.-Y. Oh, "Energy Spectrum of a Triangular Lattice in a Uniform Magnetic Field: Effect of Next-Nearest-Neighbor Hopping", Journal of the Korean Physical Society, 37, 534-539, (2000).

[S20] A. Weston, Y. Zou, V. Enaldiev, A. Summerfield, N. Clark, V. Zolyomi, A. Graham, C. Yelgel, S. Magorrian, M. Zhou, J. Zultak, D. Hopkinson, A. Barinov, T. H. Bointon, A. Kretinin, N. R. Wilson, P. H. Beton, V. I. Falko, S. J. Haigh and R. Gorbachev, "Atomic reconstruction in twisted bilayers of transition metal dichalcogenides". Nat. Nanotechnol. 15, 592-597 (2020).
